# The impact of right ventricular free wall strain on current international echocardiography guidelines for the assessment of pulmonary hypertension

**DOI:** 10.1186/s44156-026-00114-6

**Published:** 2026-06-08

**Authors:** C. J. B. Wild, G. Coghlan, A. Charalampopoulos, A. Hameed, J. Suntharalingam, R. Mackenzie Ross, D. Knight, J. Willis, J. Page, A. Gurung, M. Johnson, N. Karia, D. Oxborough, O. Peacock, D. Thompson, D. X. Augustine

**Affiliations:** 1 Royal United Hospitals Bath NHS Foundation Trust, Bath, UK; 2 Department for Health, University of Bath, Bath, UK; 3 Royal Free Hospital NHS Foundation Trust, London, UK; 4 Sheffield Teaching Hospitals NHS Foundation Trust, Sheffield, UK; 5 Golden Jubilee Hospital, Glasgow, UK; 6 Liverpool John Moores University, Liverpool, UK

**Keywords:** Pulmonary hypertension, Echocardiography, Right ventricular free wall longitudinal strain

## Abstract

**Background:**

Current guidelines define pulmonary hypertension (PH) as a mean pulmonary artery pressure (mPAP) >20mmHg at right heart catheterisation (RHC). International transthoracic echocardiography (TTE) PH guidelines recommend a multi-parameter assessment to estimate PH probability. Effectiveness of the inclusion of right ventricular free wall strain (RVFWS) has not been established using real world data.

**Study aims:**

To determine the accuracy of current European and American TTE PH guidance in detecting PH in patients attending a UK PH centre. The impact of addition of RVFWS to the efficacy of the European and American guidance was also evaluated.

**Methods:**

TTE with subsequent RHC (within 1.4 months) were undertaken in patients with suspicion of PH, referred for first time investigations. Echocardiographic variables were assessed in accordance with current European and American TTE guidance.

**Results:**

Of 549 patients assessed, 431 (79%) had RHC confirmed PH (average mPAP = 41mmHg). Sensitivity / specificity for detecting PH was calculated for the European Society of Cardiology (ESC) TTE PH recommendations (83% / 65% respectively); ESC + RVFWS (92% / 62% respectively); American Society of Echocardiography (ASE) TTE PH recommendations (89% / 49% respectively); ASE + RVFWS (96% / 36% respectively); TTE PASP > 35mmHg alone (75% / 73% respectively); TTE TRV > 2.8 m/s alone (77% / 78% respectively). Of those with RHC PH 3 (1%) subjects with a TRV > 2.8 m/s and 7 (3%) with a PASP > 35mmHg had no supporting signs of PH. Using TTE PASP > 35mmHg or TRV >2.8m/s with at least 2 abnormal TTE parameters (including RVFWS) gave similar sensitivity / specificity (74% / 79% vs 73% / 87% respectively). In those with RHC PH and TTE PASP >35mmHg or TRV >2.8m/s the significant majority had at least 2 TTE PH markers (99% & 97%). Whilst TTE PASP and RHC PASP correlation was good (r = 0.745), accuracy was poor with limits of agreements as high as 44mmHg (range = -29 to 44mmHg). In those with no measurable tricuspid regurgitation, 64% (n = 49) had RHC PH (11% of whole cohort); in those where TTE PASP <35mmHg 23% (n = 70) had RHC PH. In those felt to have an ESC PH low TTE probability 44% (n = 60) had RHC PH (14% of whole cohort). Incorporating RVFWS improved detection in those with a ESC low TTE PH probability, reducing false negatives by 43%.

**Conclusion:**

Current TTE PH algorithms lack sensitivity to detect patients with milder haemodynamic forms of PH. This can be improved with the addition of RVFWS.

**Supplementary Information:**

The online version contains supplementary material available at 10.1186/s44156-026-00114-6.

## Background

Pulmonary Hypertension (PH) is characterised by an elevated mean pulmonary artery pressure (mPAP) which exceeds 20mmHg as measured by right heart catheterisation (RHC) at rest [1]. It is caused by a variety of pathologies and untreated can be a life limiting disorder.

Whilst transthoracic echocardiography (TTE) is the main imaging tool for the detection of PH, the definitive diagnosis of PH is made by RHC. Early symptoms raising the suspicion of PH can be frequently overlooked. This has been shown to lead to an average delay to diagnosis from initial presentation, or onset of symptoms, of 2–4 years [2]. Late diagnosis negatively impacts the survival of patients for whom potential treatment options are available [3, 4]. If left untreated, PH can result in right heart failure and often death, but life expectancy in those diagnosed with PH can be significantly improved with timely identification and treatment [5]. The use of TTE in the assessment of PH has evolved with the advent of improving technology and a growing body of evidence around each measurement. Echocardiographic evaluation of tricuspid regurgitation velocity (TRV) can be used to estimate pulmonary artery systolic pressures (PASP). However, population studies have shown that TTE estimates of mPAP and PASP show insufficient precision to be used diagnostically [6–8]. Therefore, other echocardiographic measures in addition to TRV are commonly used to raise the suspicion of PH.

Whilst current international TTE guidelines on the assessment of PH [1, 9, 10] offer a practical approach, there may be additive echo measures that can help to detect milder haemodynamic forms of PH [11, 12]. Right ventricular free wall longitudinal strain (RVFWS) assesses longitudinal myocardial deformation of the free wall of the RV using speckle tracking, providing a marker of ventricular function. It is calculated by measuring the change in distance between echocardiographic speckles of the RV free wall during the cardiac cycle [13] and is displayed as a percentage. RVFWS is reduced in those with PH compared to control groups and may also provide important prognostic information [14, 15]. However, the application of RVFWS has not been previously evaluated in current international guidelines with regard to the early detection of PH.

The primary aims of this study are to evaluate the accuracy of current international echocardiographic guidelines for the assessment of PH in a large real-world cohort of patients referred for first time investigations for PH; examine the frequency of abnormal echocardiographic PH markers in a real-world population of patients referred for assessment of PH and evaluate whether incorporating RVFWS within guideline algorithms will improve the detection of PH. A secondary aim was to study the correlation and accuracy of PASP between echocardiography and RHC.

## Methods

Consecutive patients were selected from individuals referred for first-time assessment of PH at the Royal United Hospital NHS Foundation Trust, Bath, UK between 2014 and 2023. Patients were included if they had a complete dataset of RHC measurements, and their echocardiography images were sufficient to allow PH assessment as per ESC 2022 and ASE 2025 guidelines [1, 10].

The final cohort comprised 549 patients with data from TTE and RHC. Ethics approval was obtained from HRA and Health and Care Research Wales (REC reference 23/SC/0300).

### Echocardiography

TTE images were acquired using GE E9 and E95 machines, GE Healthcare (Vingmed, Trondheim, Norway). Analysis of all measurements was performed offline by a single British Society of Echocardiography (BSE) accredited operator blinded to the corresponding RHC data, using Phillips Ultrasound Workspace 2023 (Phillips Ultrasound LLC, Bothell, WA). RVFWS was acquired using TomTec Arena RV AutoStrain, and manual correction was undertaken to adjust inappropriate endocardial tracking as required. All measurements were performed in concordance with the BSE minimum dataset guidelines, BSE guidelines for the assessment of pulmonary hypertension, and BSE echocardiographic assessment of the right heart in adults guidelines [9, 16, 17].

### Audit of the European Society of Cardiology 2022 pulmonary hypertension guideline

**Fig. 1:**
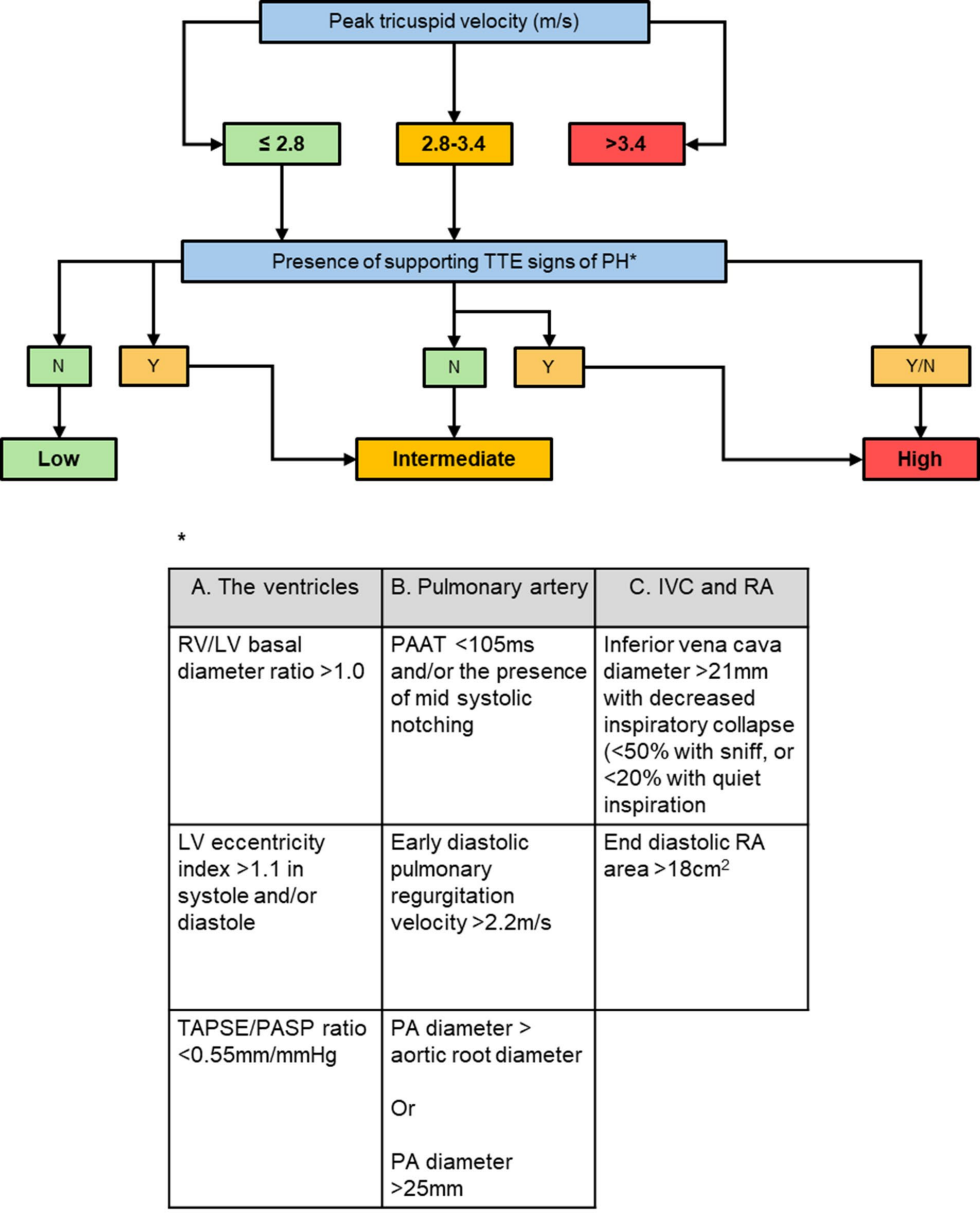
TTE PH assessment algorithm. LV: Left ventricle, RV: Right ventricle, TAPSE: Tricuspid annular plane systolic excursion, PASP: Pulmonary artery systolic pressure, PAAT: pulmonary artery acceleration time, PA: Pulmonary artery, RA: Right atrium. Adapted from the European Society of Echocardiography guidelines for PH assessment [1]

Following the release and implementation of the ESC guidelines, there has been limited research in real world populations to assess the effectiveness of these recommendations in detecting PH. Current ESC and BSE guidance [1, 9] split the supporting signs of PH into 3 groups: the right ventricle; the pulmonary artery and the right atrium / inferior vena cava. These guidelines recommend the stratification of PH TTE probability into low, intermediate, and high, using the algorithm in Fig. 1.

Following TTE analysis, patients were assigned low, intermediate, or high probability grouping according to current ESC guidelines for TTE PH assessment [1]. ESC PH TTE probability outcomes were used to separate the cohort into two categories: positive and negative. The positive group comprised individuals who had either intermediate or high echocardiographic PH probability. Patients were considered negative if their TTE PH derived probability was low.

**Fig. 2:**
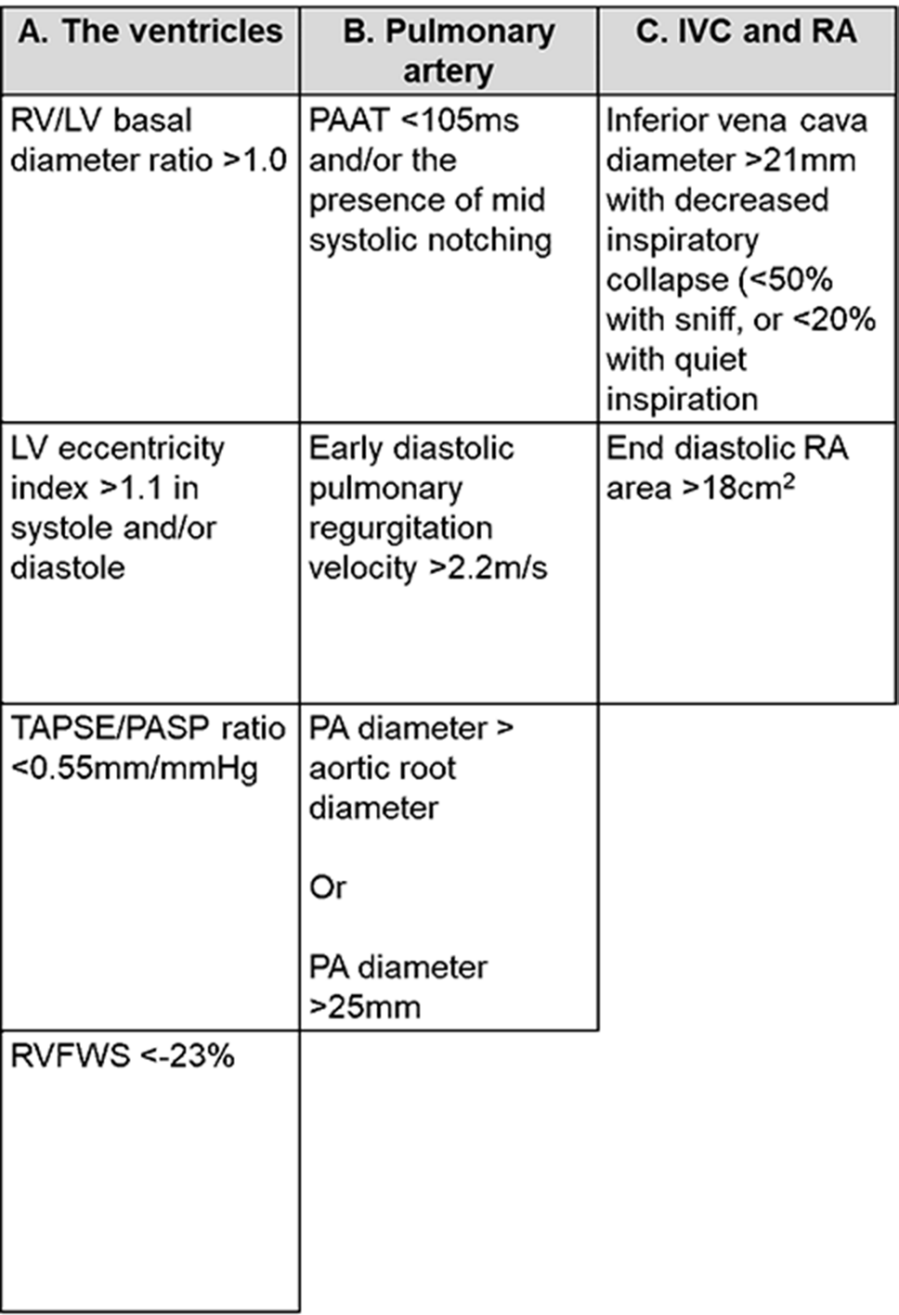
The supporting signs of PH via TTE, with the addition of abnormal RVFWS as applied in the second stage of image analysis and probability stratification. Adapted from the European Society of Echocardiography guidelines for PH assessment [1]

Abnormal RVFWS was then added to the existing ESC supporting signs of PH under category A (the ventricles), Fig. 2. The ESC PH TTE probability of the cohort was re-analysed. An abnormal RVFWS was considered a positive sign of PH and if it coincided with a positive marker from either column B or C, it would result in the patient’s probability tier being increased.

### Clinical applications of strain echocardiography: a clinical consensus statement from the American Society of Echocardiography developed in collaboration with the European Association of Cardiovascular Imaging of the European Society of Cardiology

Most recently, a joint publication from the ASE and European Association of Cardiovascular Imaging (EACVI) has reduced the threshold for abnormal RVFWS to -20% in males, and − 21% in females [18].

Analysis of the cohort has included these reduced cut-off values, in addition to the previous definition of <-23%.

### Audit of the guidelines for the echocardiographic assessment of the right heart in adults and special considerations in pulmonary hypertension: recommendations from the American Society of Echocardiography

**Fig. 3:**
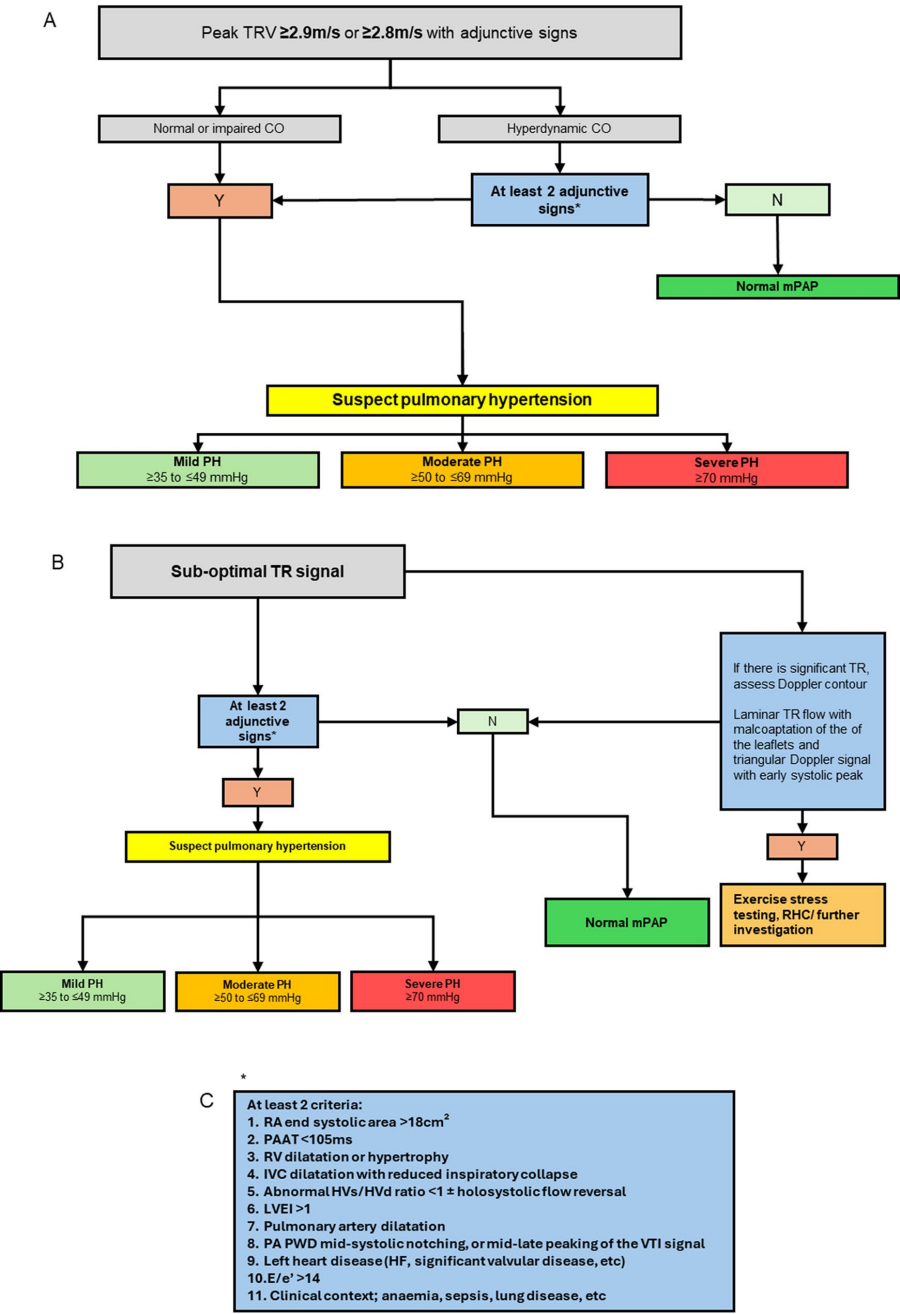
**A**: TTE PH assessment algorithm in the presence of measurable tricuspid regurgitation. **B**: TTE PH assessment algorithm in the presence of sub-optimal tricuspid regurgitation imaging. **C**. Adjunctive signs of PH. LV: Left ventricle, RV: Right ventricle, TAPSE: Tricuspid annular plane systolic excursion, PASP: Pulmonary artery systolic pressure, PAAT: pulmonary artery acceleration time, PA: Pulmonary artery, RA: Right atrium. Adapted from the guidelines for the echocardiographic assessment of the right heart in adults and special considerations in pulmonary hypertension: recommendations from the American Society of Echocardiography [10]

In 2025, the American Society of Echocardiography (ASE) has released guidelines for the assessment of PH via TTE (Fig. 3) [10]. Here, assessment of resting TRV in addition to at least two adjunctive echocardiographic signs is used to suggest PH. Furthermore, echocardiographic categorisation of the severity of PH has been recommended (mild PH PASP ≥ 35 to ≤ 49mmHg; moderate PH ≥ 50 to ≤ 69mmHg; severe PH ≥ 70 mmHg).

The cohort was then analysed using the ASE criteria (Fig. 3) [10] to assess for the presence of PH and haemodynamic severity. Here, the TRV is initially assessed and ASE flowchart applied. Ultimately, PH is then classified into severity groups based on the TTE PASP (mild PH ≥ 35 to ≤ 49mmHg; moderate PH ≥ 50mmHg to ≤ 69mmHg; severe PH ≥ 70mmHg).

### Statistical analysis

Continuous data is expressed as mean ± standard deviation. Receiver Operator Curve analysis was used to assess sensitivity and specificity of optimal cut-offs. The cohort was tested for normalcy using a Shapiro-Wilk test (p = < 0.001, C.I. = 95%). Independent T-test was used to derive differences in parameters between patients with and without elevated mPAP. Sensitivity and specificities of the existing ESC PH TTE algorithm for the cohort, with and without RVFWS was achieved via crosstabulation (IBM, SPSS Statistics V29.0.1.1., 2023).

## Results

Characteristics of the 549 individuals included in the study are shown in Table 1.

The cohort was predominantly female (62%), with mean age of 64 ± 14 years. The mean elapsed time between initial TTE and corresponding RHC was 44 ± 61 days.

**Table 1:** Characteristics for 549 individuals referred for first-time assessment of PH at a national UK PH centre

Number of patients	549
Age (years)	64 ± 14
Male/female (%)	208/341 (38/62)
Height (cm)	166 ± 17
Weight (kg)	82 ± 21
Dubois BSA (m²)	1.9 ± 0.3
Time to RHC (days)	44 ± 61

Data is expressed as mean±SD, or as a number depicting frequency (percentage)

### ASE 2025 guideline audit

All 549 patients were included in the analysis. Measured values specific to the ASE algorithm for screening patients with suspected pulmonary hypertension are shown in Table 2.

**Table 2:** Echocardiographic characteristics

Parameter	Whole cohort (*n* = 549)	PH (*n* = 431)	No PH (*n* = 118)	TRV > 2.9 m/s or > 2.8 m/s with adjunctive signs (*n* = 319)	TRV < 2.8 m/s (*n* = 153)
Cardiac output (L/min)	4.6 ± 1.7	4.5 ± 1.5	5.2 ± 2.3	4.5 ± 1.6	4.7 ± 1.9
RA > 18 cm² (*n* = 522)	282 (51%)	254 (59%)	28 (24%)	201 (63%)	53 (35%)
PAAT < 105ms (*n* = 516)	375 (68%)	325 (75%)	50 (43%)	252 (79%)	82 (54%)
RV dilatation and/or hypertrophy* (*n* = 507)	266 (49%)	229 (53%)	37 (31%)	186 (58%)	52 (34%)
IVCd > 2.1 cm with collapse < 50% (62)	62 (11.2%)	62 (14%)	0	50 (16%)	10 (7%)
EId > 1 (*n* = 457)	209 (38%)	205 (47%)	14 (12%)	168 (53%)	30 (20%)
EIs > 1 (*n* = 447)	222 (40%)	194 (45%)	18 (15%)	172 (54%)	37 (24%)
PA diameter > 2.5 cm (*n* = 444)	118 (22%)	104 (24%)	14 (12%)	86 (27%)	20 13%)
PV mid systolic notch or late VTI peak	63 (12%)	57 (13%)	6 (5%)	51 (16%)	6 (4%)
Left heart disease (*n* = 481)	128 (23%)	109 (25%)	19 (16%)	79 (25%)	34 (22%)
E/e’ >14 (*n* = 439)	37 (7%)	33 (8%)	4 (3%)	28 (9%)	8 (5%)

Data is presented as mean ± SD, or as a number depicting frequency and a percentage. RA right atrium, PAAT pulmonary artery acceleration time, RV right ventricle, EI eccentricity index, strain, PV pulmonary valve, PR pulmonary regurgitation, PA pulmonary artery, AR aortic root, IVC inferior vena cava. *This row represents RV dilatation only, as none of this cohort were found to have measured RV hypertrophy

Abnormal markers were consistently more prevalent in those with PH than without. The highest proportion of abnormal markers related specifically to the chambers of the right heart, with 53% of those with PH presenting with RV dilatation and 59% with RA dilatation. RV hypertrophy was not identified within the cohort.

Overall, the ASE criteria for PH screening and early detection of PH gave a sensitivity and specificity of 89% and 49%, respectively.

### ESC 2022 TTE PH guideline audit

**Fig. 4:**
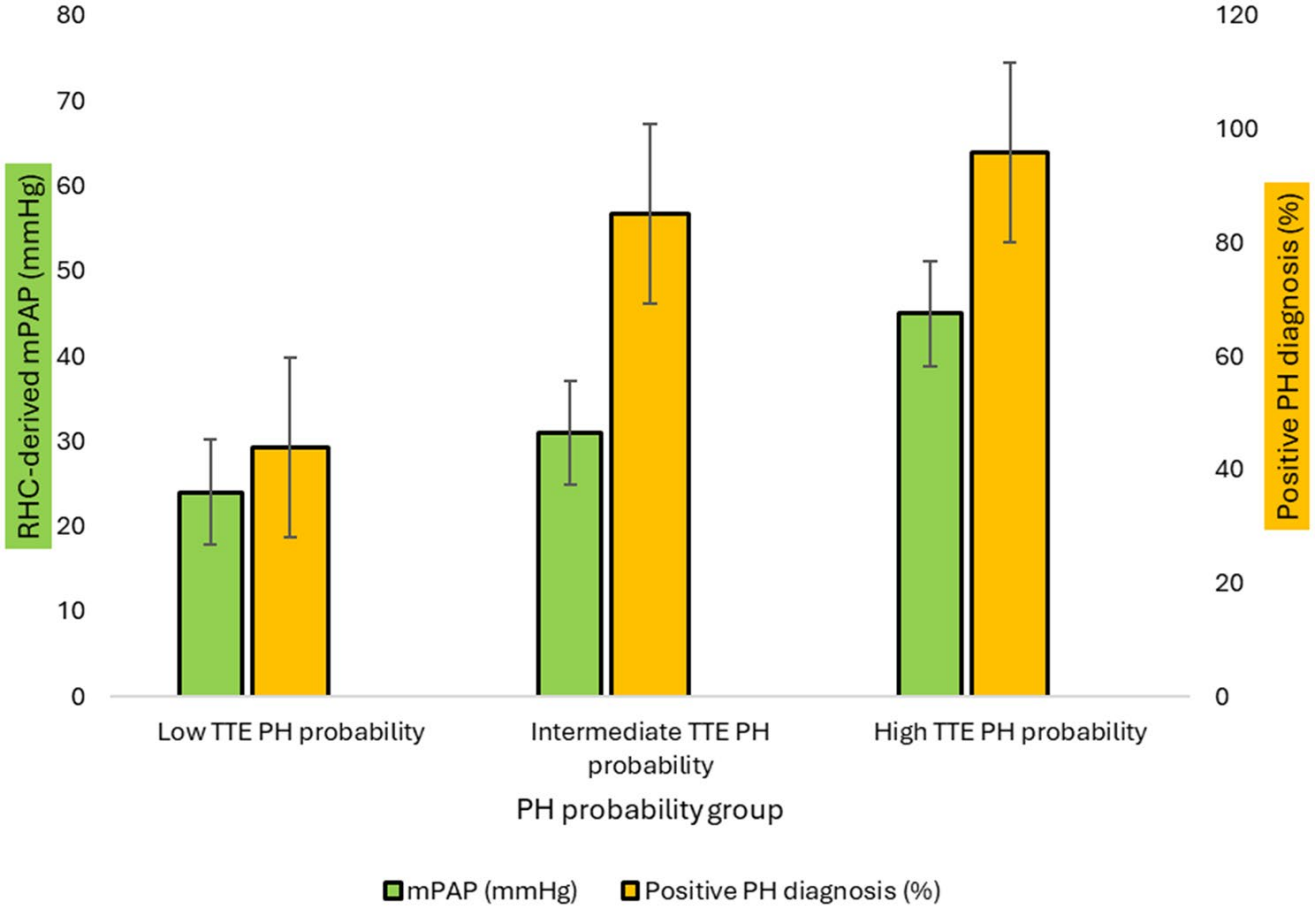
Percentage of 549 total patients with mPAP > 20mmHg, organised by ESC TTE probability

From the cohort of 549 patients, 51% (*n* = 283) had a high probability of PH by ESC criteria, 24% (*n* = 130) an intermediate probability and 25% (*n* = 136) a low TTE probability of PH. Those patients with a higher echo probability of PH had worse RHC haemodynamics (high TTE PH probability mPAP = 45 ± 12mmHg; intermediate TTE PH probability mPAP 31 ± 11mmHg; low TTE PH probability mPAP 23 ± 8mmHg) (Fig. 4).

96% (*n* = 271) of those in the high probability group and 77% (*n =* 100) of those in the intermediate probability groups had a RHC mPAP > 20mmHg. 44% (*n* = 60) of those within the low probability group had a RHC mPAP > 20mmHg (Table 3).

**Table 3:** PH characteristics

**A. Positive PH presence by TTE, and formal positive diagnosis by gold standard RHC**				
	**mPAP > 20mmHg (%)**	**mPAP < 20mmHg (%)**		
ESC outcome	412 (75)	137 (25)		
ASE outcome	378 (88)	53 (45)		
RHC outcome	431 (79)	118 (21)		
**B. Positive PH diagnosis by ESC TTE PH probability group**				
	**mPAP > 20mmHg (%)**	**mPAP < 20mmHg (%)**	**mPAP (± SD)**	**Total (%)**
High	271 (96)	12 (4)	45 ± 12	283 (51)
Intermediate	100 (77)	30 (23)	31 ± 11	130 (24)
Low	60 (44)	76 (56)	23 ± 8	136 (25)

Data is presented as mean *±* SD, or as a number depicting frequency, and a percentage. A: Frequency of positive (intermediate and high) and negative (low) PH probability via TTE. B: Frequency of formal PH diagnosis as derived by mPAP > 20mmHg at gold standard RHC, with mean PA pressure grouped by probability

The sensitivity and specificity of the ESC PH TTE probability algorithm was 83% and 65% (AUC = 0.76; C.I. = 95%, 0.69–0.82) respectively.

RVFWS was measurable in 390 patients (mean = -18 ± 8.4). Applied across the entire cohort, a cut off of -23% produced a sensitivity and specificity of 80% and 60%, respectively (AUC = 0.77; C.I. = 95%, 0.71–0.82). The addition of RVFWS improved the sensitivity and specificity for the detection of PH in the low echo probability group from 44% to 56% to 81% and 63% respectively (AUC = 0.74; C.I. = 95%, 0.64–0.85) (Supplementary Fig. 2).

Using the most recent [18] RVFWS cut-off for males of -20% gave a sensitivity and specificity of 75% and 70%, respectively across the entire cohort. Within the low probability group this reduced cut-off demonstrates a small increase in sensitivity (50%) and a marked increase in specificity (88%).

In females, the reduced cut-off of 21% gave a sensitivity of 70% and a specificity of 67%. Within the low probability group, a sensitivity and specificity of 40% and 93% was seen.

### Echocardiographic markers of PH

**Fig. 5:**
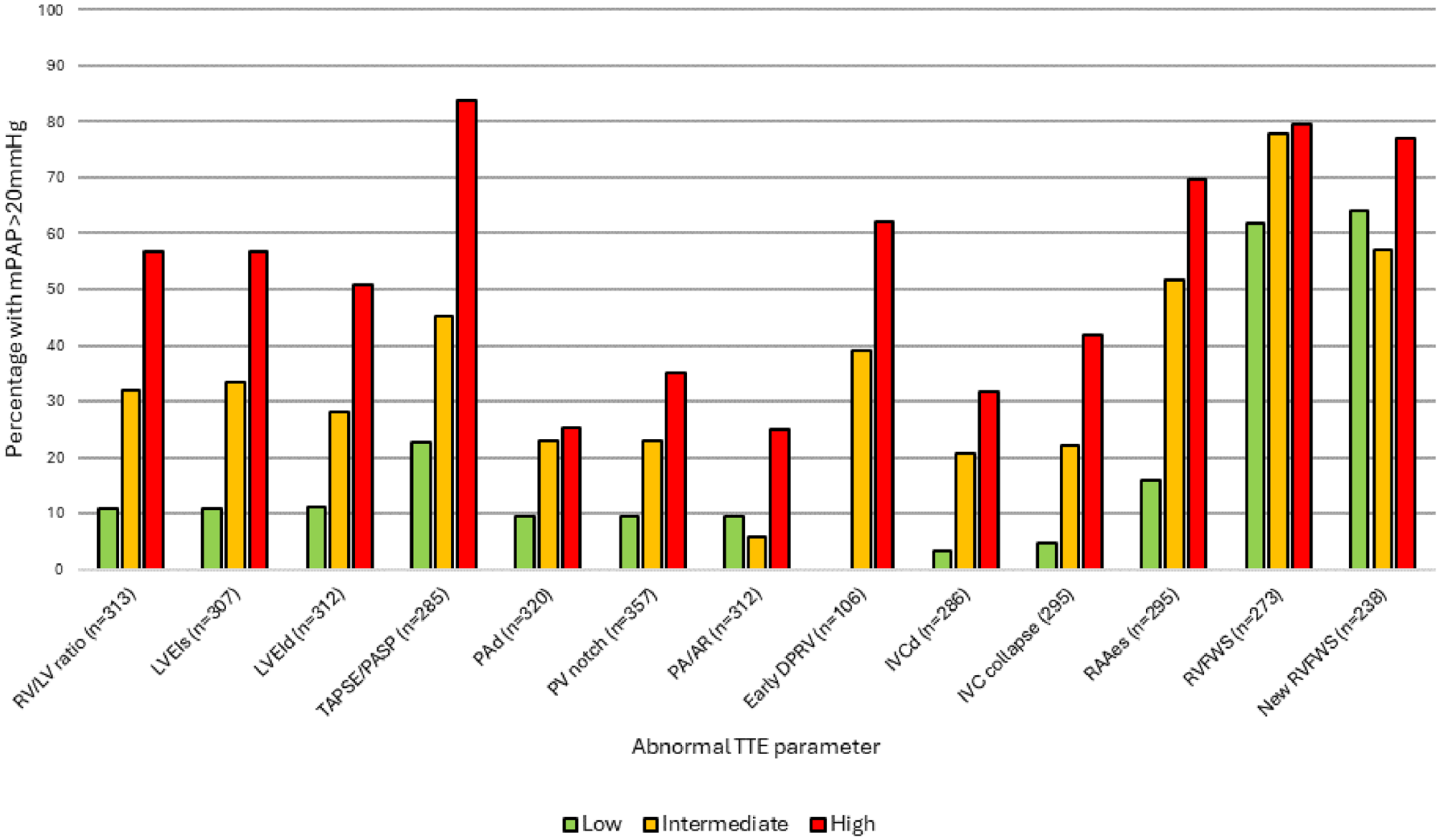
Frequency of abnormal TTE PH probability markers in those with Low, Intermediate & High ESC probability, with mPAP > 20mmHg as measured by gold standard RHC. Red: High TTE probability of PH. Orange: Intermediate TTE probability of PH. Green: Low TTE probability of PH

Figure 5 depicts the frequency of abnormal markers across the three TTE PH probability tiers.

Frequency of which echo parameters were measurable; receiver operating characteristics and mean values for those with and without PH are shown in Table 4. The highest frequency of abnormal parameters was seen in the high ESC PH TTE probability group. Pulmonary artery / aortic root ratio had low yield, < 30% in any of the probability groups. Reduced RVFWS and PAAT were the only measures that were present in at least 60% of the intermediate and high TTE echo probability groups. RVFWS was the only measure present in at least 50% of each of the TTE PH probability groups.

**Table 4:** Echocardiographic characteristics

		RHC diagnosis					
	TTE parameter	PH (± SD)	No PH (± SD)	*P* value	Optimum cut off	AUC	% Measured
	TRV peak (m/s)	3.1 ± 1.4	2.4 ± 0.71	< 0.001	2.7	0.84	85
A. The Ventricles	RV/LV ratio	1.0 ± 0.3	0.8 ± 0.22	< 0.001	0.7	0.71	83
	End-systolic EI	1.1 ± 0.4	0.8 ± 0.3	< 0.001	1.0	0.73	83
	End-diastolic EI	1.1 ± 0.5	0.8 ± 0.3	< 0.001	1.0	0.74	84
	TAPSE/PASP ratio	0.70 ± 1.45	1.32 ± 1.62	< 0.001	0.55	0.81	82
	RVFWS (-23%)						
	*Low*					0.75	
	*Low & intermediate*					0.74	
	New RVFWS (Male: -20%) *Low*					0.78	
	New RVFWS (Female: -21%) *Low*					0.76	
	*Whole Cohort*	-17.1 ± 6.5	-23.4 ± 5.7	< 0.001	-23	0.77	71
B. Pulmonary artery	PAAT (ms)	81.7 ± 30	108.2 ± 35.5	< 0.001	96	0.74	93
	Early diastolic PR (m/s)	1.3 ± 1.1	0.7 ± 0.9	< 0.001	0.9	0.65	29
	Mid-systolic notch	63	3	N/A	N/A	N/A	12
	PA diameter (cm)	2.0 ± 0.1	1.9 ± 0.8	0.253	2.2	0.56	80
	PA/AR ratio	0.7 ± 0.3	0.7 ± 0.3	0.98	0.8	0.59	90
C. Inferior vena cava and right atrium	IVC diameter	1.8 ± 1.2	1.4 ± 0.7	0.001	1.6	0.64	79
	IVC collapse < 50%	55.7 ± 26.2	57.6 ± 30.8	0.543	60	0.55	76
	RA area (cm²)	20.6 ± 8.2	15.5 ± 6.1	< 0.001	16	0.70	94

Data is presented as mean ± SD, or a percentage. RV right ventricle, LV left ventricle, EI eccentricity index, TAPSE tricuspid annular plane systolic excursion, RVFWS right ventricular free wall strain, PAAT pulmonary artery acceleration time, PV pulmonary valve, PR pulmonary regurgitation, PA pulmonary artery, AR aortic root, IVC inferior vena cava, RA right atrium

Table 4 describes the echocardiographic variables measured, including the mean values together with optimal cut offs and AUCs derived from ROC curves (Supplementary Figs. 3a, b, c).

Echocardiographic markers were measurable in > 80% of the cohort and were significantly worse in those with PH compared to those without (p = < 0.001), Table 4. In those patients with confirmed RHC PH, 3% (*n* = 11) had no echocardiographic measures present; 7% (*n* = 29) had 1 echocardiographic measure present; 12% (*n* = 53) had 2 echocardiographic measures present and 78% (*n* = 338) had > 2 echocardiographic measures present.

Within the pulmonary artery echocardiographic markers, PAAT and PR end diastolic velocity were significantly different in those with PH compared to those without (p = < 0.001).

PA diameter (*p* = 0.253) and PA/AR ratio (*p* = 0.98) were comparable in those with PH and those without, with no significant difference. These measures also demonstrated poor AUC (0.556 and 0.584 respectively, Supplementary Fig. 2). Mid-systolic notching was present within only 12% of the total cohort, but of these, 95% were positive for PH at RHC.

### RVFWS

ROC curve analysis demonstrated a reasonable AUC of 0.77 with an optimal cut off of < -23%. Using the established cut off of <-23% in the low probability group gave a sensitivity of 81% and a specificity of 63% (C.I. = 95%, 0.64–0.85). Applied across the entire cohort, a cut off of -23% produced a sensitivity and specificity of 80% and 60%, respectively (C.I. = 95%, 0.72–0.83) (Supplementary Fig. 1).

Reanalysis of the cohort with the addition of RVFWS <-23% to the ASE algorithm and adjunctive signs of PH gave a sensitivity of 96% and specificity of 36%.

Reanalysis of the cohort with the addition of RVFWS to the ventricular column of the ESC supporting signs of PH demonstrated a sensitivity of 92% and a specificity of 62%. False negatives were reduced from 14 patients, to 8 (43% reduction). Two patients with mPAP < 20mmHg were upgraded from low probability to intermediate and provided a false positive, accounting for this reduction in specificity (2%).

Using the most recent cut-off values for abnormal RVFWS as outlined in a joint publication by the ASE and EACVI [18] AUC in males was 0.79, and in females 0.76. Repeat analysis of the whole cohort with the RVFWS cut-off reduced to -20% in males, and − 21% in females with the ESC algorithm saw sensitivity increased to 93%, and specificity reduced to 60%.

Applying the same cut-offs to the ASE algorithm gave a sensitivity and specificity of 94% and 37%, respectively (Table 5).

**Table 5:** Calculated sensitivity and specificity values of the ESC guidelines for the echocardiographic assessment of PH in identifying patients with a mean PA pressure greater than 20mmHg. Results represent both the existing algorithm, and utilising the addition of abnormal RVFWS in the ventricular column of the supporting signs of PH

		mPAP < 20mmHg (%)	mPAP > 20mmHg (%)
ESC outcome	Negative	76 (64)	61 (14)
	Positive	36 (36)	370 (86)
ESC+RVFWS outcome	Negative	67 (62)	32 (8)
	Positive	42 (38)	369 (92)
ASE outcome	Negative	44 (49)	41 (11)
	Positive	46 (51)	346 (89)
ASE+RVFWS outcome	Negative	33 (36)	17 (4)
	Positive	59 (64)	385 (96)
ESC + New RVFWS outcome	Negative	50 (60)	26 (7)
	Positive	34 (40)	361 (93)
ASE + New RVFWS outcome	Negative	33 (37)	23 (6)
	Positive	57 (63)	364 (94)

### TTE-derived pulmonary artery systolic pressure

70% (*n* = 382) of the overall cohort had images sufficient to estimate non-invasive RA pressure and were eligible for this sub-analysis (Table 6). TTE RAP had poor correlation with RHC RAP (*R* = 0.39) with a significant difference in means; 6mmHg for TTE vs. 10mmHg for RHC, p = < 0.05.

Non-invasive RA pressure was measured using IVC diameter and inspiratory collapse percentage. PASP was then estimated using a combination of RA pressure and tricuspid regurgitant peak gradient.

68% (*n* = 260) patients had a TTE derived PASP > 35mmHg. 77% of those with PH at RHC had a non-invasive estimate of PASP in excess of 35mmHg.

**Table 6:** Means & parameters by PH diagnosis

	**Whole cohort**	**No PH**	**PH**
TRVPG (mmHg)	46 ± 24	25	51 ± 24
TTE PASP (mmHg)	51 ± 27	27 ± 13	56 ± 26
IVC diameter (cm)	1.9 ± 1	1.6 ± 0.4	1.9 ± 1.1
IVC collapse (%)	60 ± 22	67 ± 18	59 ± 23
RHC mPAP (mmHg)	37 ± 15	17 ± 2	41 ± 13
	**Whole cohort (%)**	**No PH (%)**	**PH (%)**
Est RAP = 3mmHg*	216 (57)	51 (71)	165 (53)
Est RAP = 8mmHg*	104 (27)	20 (28)	84 (27)
Est RAP = 15mmHg*	62 (16)	1 (1)	60 (19)
TTE PASP > 35mmHg	260 (68)	20 (28)	239 (77)
TTE PASP ≤ 35mmHg	122 (32)	52 (72)	70 (23)

Data is presented as mean ± SD, or frequency and a percentage. RHC right heart catheter, TRVPG tricuspid regurgitant velocity peak gradient, IVC inferior vena cava, RAP right atrial pressure

**Fig. 6:**
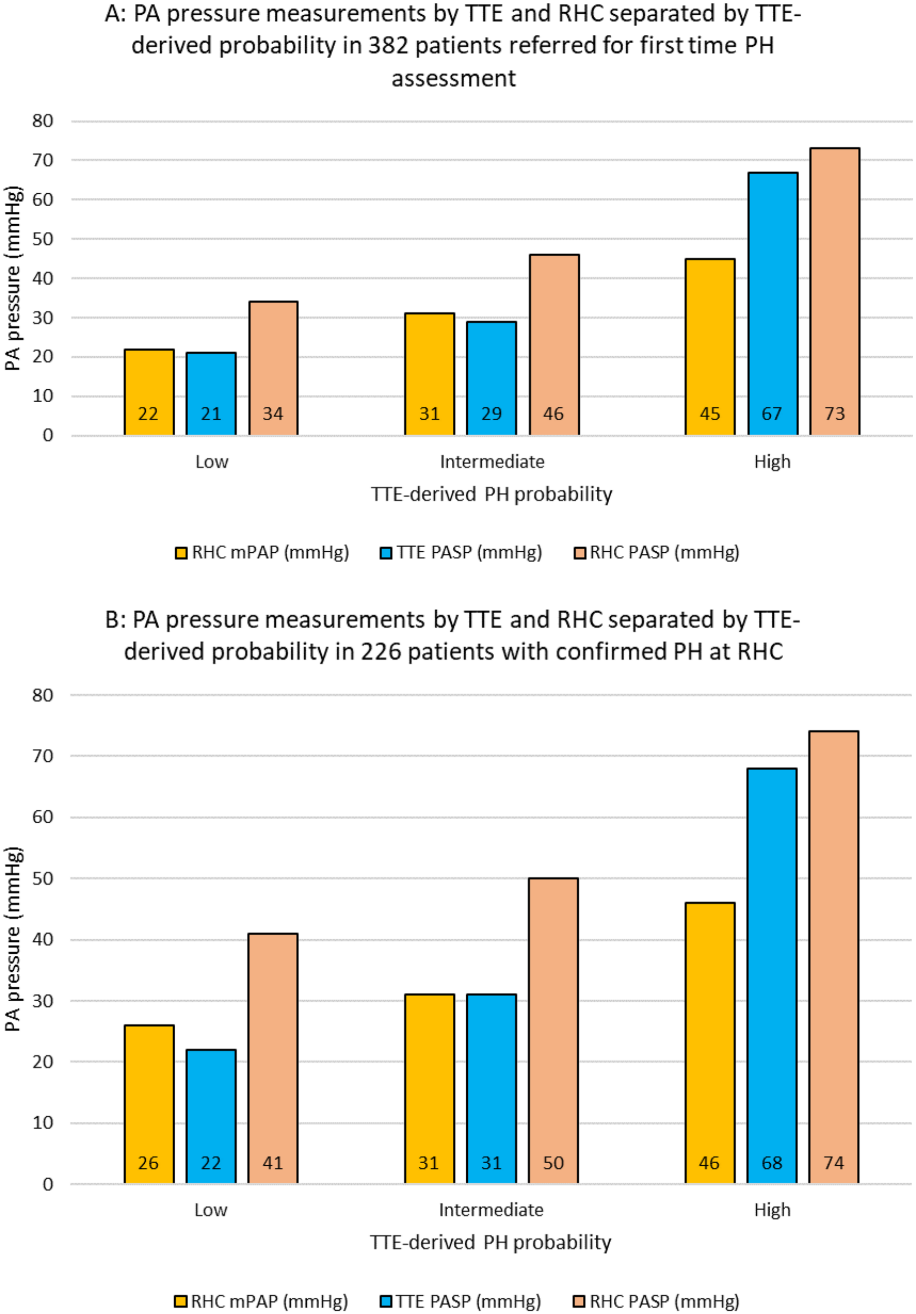
**A**: PA pressure measurements by TTE and RHC separated by TTE-derived probability of PH. **B**: PA pressure measurements by TTE and RHC in patients with confirmed PH, separated by TTE-derived probability of PH

Figure 6 depicts the mean RHC mPAP values obtained for each of the TTE PH probability groups together with PASP pressure estimates obtained from RHC and TTE in the whole group as well as in patients with confirmed PH at RHC. Mean values for TTE-derived PASP are significantly lower than whose obtained by RHC in all echo PH probability groups (p = < 0.05). Across the whole cohort the mean TTE PASP (50mmHg) was significantly lower than that obtained by RHC (60mmHg) (p = < 0.05).

**Fig. 7:**
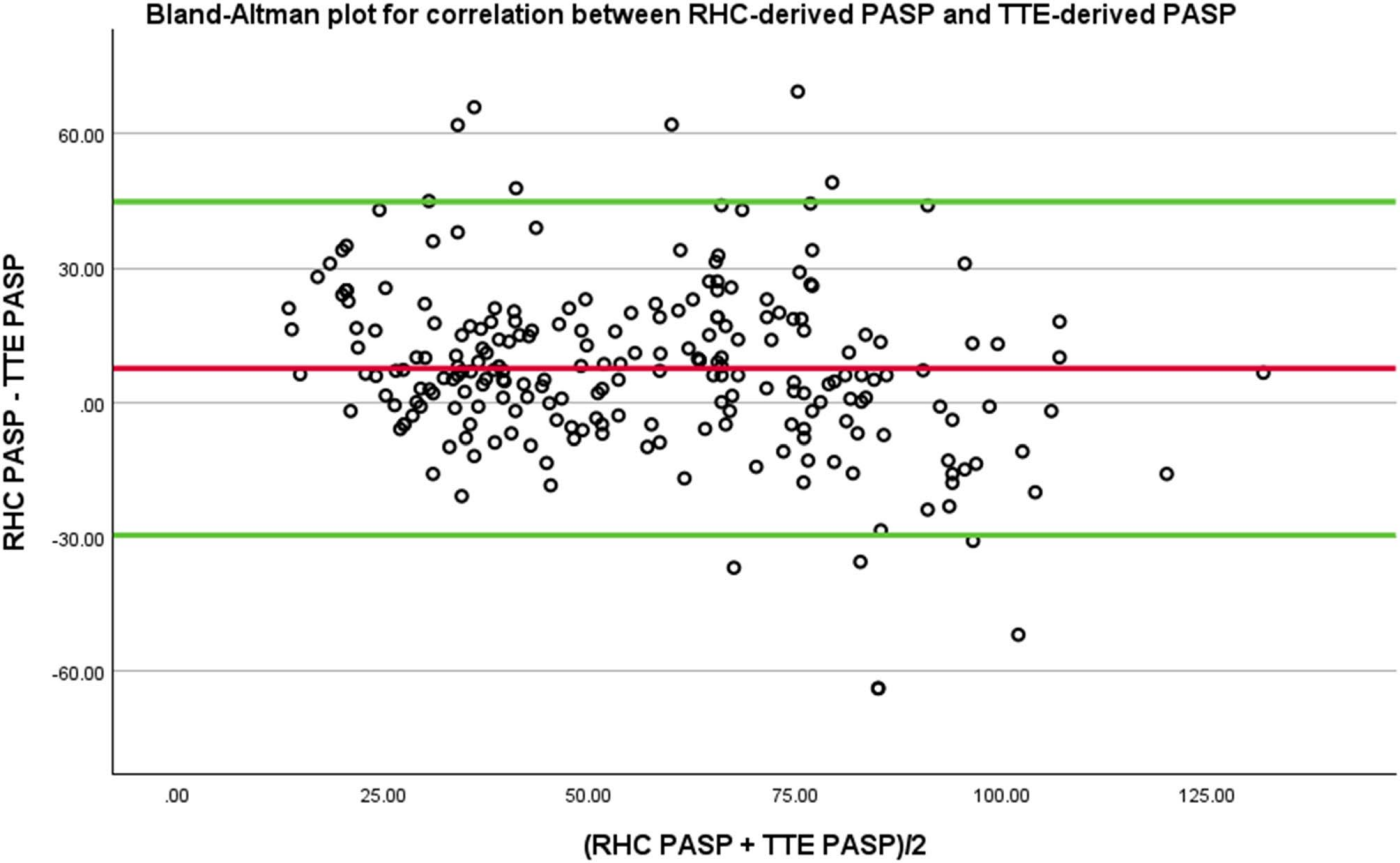
Bland-Altman plot for correlation between RHC-derived PASP and TTE-derived PASP estimates

There is good PASP correlation between TTE and RHC (*R* = 0.745, p = < 0.05). However, whilst Bland Altman analysis demonstrated moderate bias (7mmHg), there were poor limits of agreement with discrepancies of up to 44mmHg (range − 29 to 44mmHg) (Fig. 7).

**Fig. 8:**
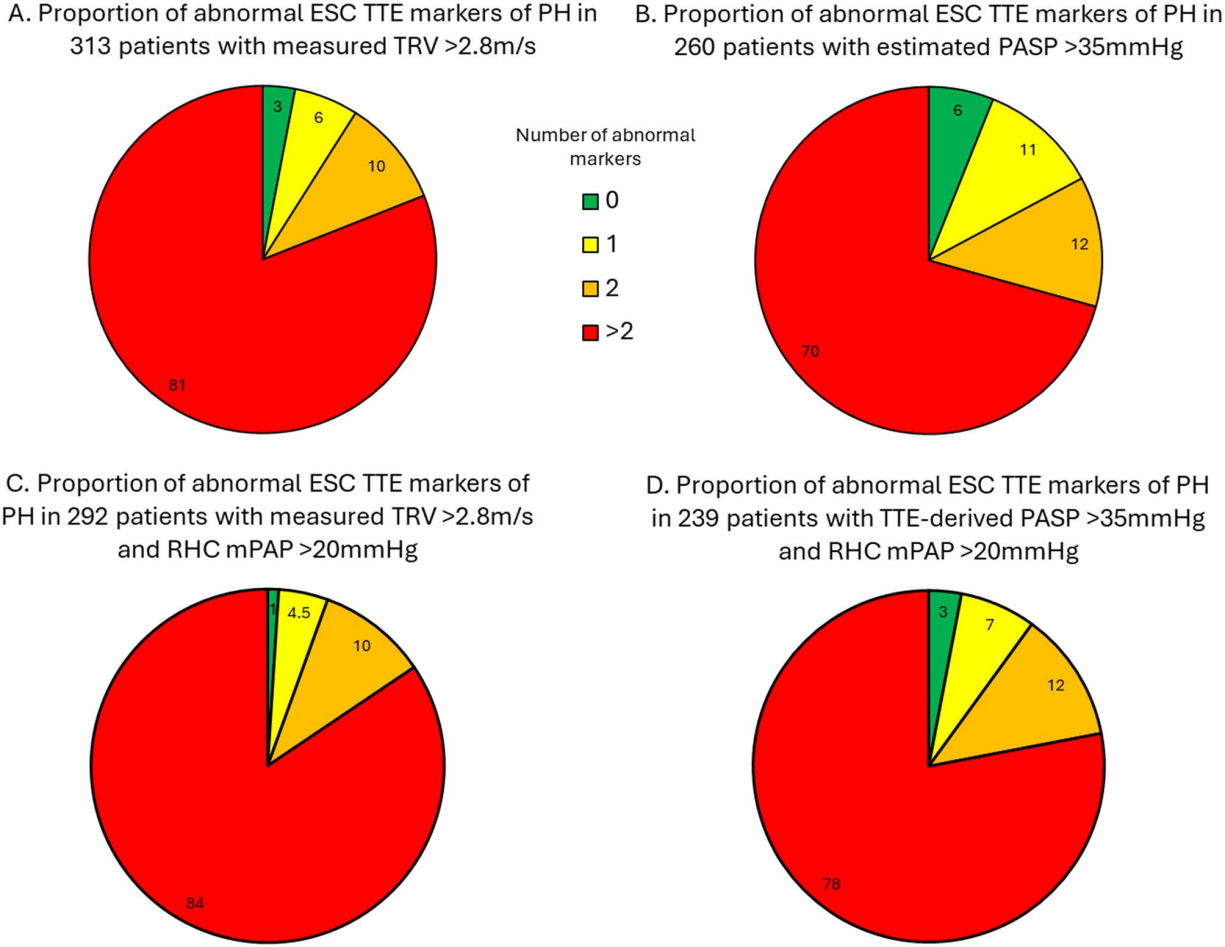
Charts showing the percentage proportion of abnormal TTE markers of PH in patients referred for first time investigation for PH with **A**: a measured TRV > 2.8 m/s and any RHC mPAP, **B**: an echo derived PASP > 35mmHg and any RHC mPAP, **C**: a measured TRV > 2.8 m/s and RHC mPAP > 20mmHg. **D**: an echo derived PASP > 35mmHg and RHC mPAP > 20mmHg

Across the cohort of 382, an echocardiographic PASP value > 35mmHg identified RHC PH in 239 patients (62%). In those with echocardiographic PASP ≤ 35mmHg (*n* = 122), 70 (23%) had RHC PH. Almost all of this cohort was found to have 1 or more ESC TTE markers of PH, Fig. 8. In the absence of any ESC TTE markers of PH the sensitivity and specificity of PASP > 35mmHg to detect PH is 75% / 73% and that for TRV > 2.8 m/s is 77% / 78%.

In patients with a RHC derived mPAP greater than 20mmHg, 84% of patients with a measurable TRV > 2.8 m/s had 3 or more abnormal ESC supporting signs of PH, and 78% of those with a TTE-derived PASP > 35mmHg had 3 or more abnormal ESC supporting signs of PH. In this group with PH, only 7 (3%) patients with TTE-derived PASP > 35mmHg and RHC mPAP > 20mmHg had no abnormal ESC supporting signs of PH. This was only 3 (1%) in patients with TRV > 2.8 m/s and RHC mPAP > 20mmHg.

Combining echocardiographic PASP > 35mmHg with one echo parameter of PH as outlined by the ESC guidelines gave sensitivity / specificity for RHC PH detection of 79 / 73%. Combining echocardiographic PASP > 35mmHg with at least two echo parameters of PH gave sensitivity/specificity for RHC PH detection of 76 / 82%. Combining echocardiographic PASP > 35mmHg with at least 3 echocardiographic parameters gave sensitivity / specificity of 71 / 84%.

Combining echocardiographic PASP > 35mmHg with one echo parameter of PH including RVFWS gave sensitivity / specificity for RHC PH detection of 76 / 67%. Combining echocardiographic PASP > 35mmHg with at least two echo parameters of PH including reduced RVFWS gave sensitivity / specificity for RHC PH detection of 74 / 79%. Combining echocardiographic PASP > 35mmHg with at least 3 echocardiographic parameters including reduced RVFWS gave sensitivity / specificity of 70 / 84%.

Combining echocardiographic peak TRV > 2.8 m/s with one echo parameter of PH gave sensitivity / specificity for RHC PH detection of 76 / 82%. Combining echocardiographic peak TRV > 2.8 m/s with at least two echo parameters of PH gave sensitivity / specificity for RHC PH detection of 72 / 88%. Combining echocardiographic peak TRV > 2.8 m/s with at least 3 echocardiographic parameters gave sensitivity / specificity of 65 / 91%.

Combining echocardiographic peak TRV > 2.8 m/s with one echo parameter of PH including reduced RVFWS gave sensitivity / specificity for RHC PH detection of 76 / 81%. Combining echocardiographic peak TRV > 2.8 m/s with at least two echo parameters of PH including reduced RVFWS gave sensitivity / specificity for RHC PH detection of 73 / 87%. Combining echocardiographic peak TRV > 2.8 m/s with at least 3 echocardiographic parameters including reduced RVFWS gave sensitivity / specificity of 67 / 91% (Table 7).

**Table 7:** Calculated sensitivity and specificity values for TTE TRV and PASP in detecting PH both as standalone measurements, in conjunction with the existing ESC supporting signs of PH, and with RVFWS as an additional marker

		mPAP < 20mmHg (%)	mPAP > 20mmHg (%)
TRV > 2.8 m/s	Negative	70 (78)	87 (22)
	Positive	20 (22)	293 (77)
TRV > 2.8 m/s + 1 marker	Negative	74 (82)	91 (24)
	Positive	16 (18)	286 (76)
TRV > 2.8 m/s + ≥ 2 markers	Negative	79 (88)	104 (27)
	Positive	11 (12)	271 (72)
TRV > 2.8 m/s > 2 markers	Negative	82 (91)	134 (36)
	Positive	90 (9)	376 (64)
TRV > 2.8 m/s + 1 marker inc RVFWS	Negative	74 (82)	91 (24)
	Positive	16 (18)	286 (76)
TRV > 2.8 m/s + ≥ 2 markers inc RVFWS	Negative	79 (88)	104 (28)
	Positive	11 (12)	271 (72)
TRV > 2.8 m/s > 2 markers inc RVFWS	Negative	82 (91)	134 (36)
	Positive	8 (9)	242 (64)
PASP > 35mmHg	Negative	40 (73)	82 (25)
	Positive	15 (27)	245 (75)
PASP > 35mmHg + 1 marker	Negative	47 (73)	63 (20)
	Positive	17 (27)	238 (79)
PASP > 35mmHg + ≥ 2 markers	Negative	53 (82)	70 (23)
	Positive	11 (17)	231 (77)
PASP > 35mmHg > 2 markers	Negative	54 (84)	88 (29)
	Positive	10 (16)	213 (71)
PASP > 35mmHg + 1 marker inc RVFWS	Negative	32 (67)	75 (24)
	Positive	16 (33)	243 (76)
PASP > 35mmHg + ≥ 2 markers inc RVFWS	Negative	38 (79)	82 (26)
	Positive	10 (21)	236 (74)
PASP > 35mmHg > 2 markers inc RVFWS	Negative	41 (84)	94 (30)
	Positive	8 (16)	222 (70)

Data is presented as frequency and a percentage. PASP Pulmonary artery systolic pressure, TRV tricuspid regurgitant velocity, RVFWS Right ventricular free wall strain

**Table 8:** Calculated sensitivity and specificity values for TTE TRV and PASP in detecting PH in conjunction with the existing ESC supporting signs of PH, and with the new definition of abnormal RVFWS as an additional marker

		mPAP < 20mmHg (%)	mPAP > 20mmHg (%)
TRV > 2.8 m/s + 1 marker inc new RVFWS	Negative	73 (81)	92 (24)
	Positive	17 (19)	288 (75)
TRV > 2.8 m/s + ≥ 2 markers inc new RVFWS	Negative	24 (73)	130 (41)
	Positive	9 (27)	184 (59)
TRV > 2.8 m/s > 2 markers inc new RVFWS	Negative	27 (82)	148 (47)
	Positive	6 (18)	166 (53)
PASP > 35mmHg + 1 marker inc new RVFWS	Negative	12 (19)	8 (2)
	Positive	50 (81)	316 (98)
PASP > 35mmHg + ≥ 2 markers inc new RVFWS	Negative	30 (48)	22 (7)
	Positive	32 (52)	302 (93)
PASP > 35mmHg > 2 markers inc new RVFWS	Negative	43 (70)	58 (18)
	Positive	62 (31)	324 (82)

Data is presented as frequency and a percentage. PASP Pulmonary artery systolic pressure, TRV tricuspid regurgitant velocity, RVFWS Right ventricular free wall strain

When the same parameters are applied using the recently-proposed RVFWS cut offs for males and females, all subgroups using peak TRV > 2.8 m/s, sensitivity and specificity were reduced (Table 8).

When using PASP > 35mmHg, sensitivity was consistently increased, but specificity fell significantly when 1 or 2 positive supporting sings were included. Overall, sensitivity and specificity suffer across all categories (Table 9), except PASP > 35mmHg with greater than two positive supporting signs, which sees a higher sensitivity than the previous definition of abnormal RVFWS, but a reduction in specificity (Sens / spec = 82% / 70%).

**Table 9:** Calculated sensitivity and specificity values for TTE TRV and PASP in detecting PH in conjunction with the existing ESC supporting signs of PH

	RVFWS <-23%		RVFWS <-20% in males & <-21% in females	
	Sensitivity (%)	Specificity (%)	Sensitivity (%)	Specificity (%)
TRV + 1 marker inc RVFWS	76	82	75	81
TRV ≥ 2 markers inc RVFWS	72	88	59	73
TRV > 2 markers inc RVFWS	64	91	53	82
PASP + 1 marker inc RVFWS	76	67	98	19
PASP ≥ 2 markers inc RVFWS	74	79	93	48
PASP > 2 markers inc RVFWS	70	84	82	70

Data is presented as a percentage. PASP Pulmonary artery systolic pressure, TRV tricuspid regurgitant velocity, RVFWS Right ventricular free wall strain

## Discussion

### Multiparametric TTE assessment of PH

PH is a life-threating disorder, and the progression of the condition means that early recognition and treatment is a vital determinant of long-term prognosis [5]. TTE is commonly the first line imaging for investigating the presence of PH. Both the ESC and ASE PH echo guidance [1, 10] recommend a multi parameter approach to evaluate the likelihood of PH being present.

This study has shown that both ESC and ASE echo recommendations have good sensitivity (83% and 89% respectively) but moderate specificity (65% and 49% respectively) for identifying individuals at risk of PH. The best sensitivity was seen when using the ESC algorithm with the addition of RVFWS <-23% (92%), with comparable specificity (62%).

Most optimal echo measures corresponded well with current international guidelines for cut offs within this cohort. Acceptable AUCs were obtained for the majority of echo markers with the exception of early diastolic PR velocity (0.65), PA/AR ratio (0.59) PA diameter (0.56), and IVC diameter measurement (0.64) and IVC collapse (0.55).

Using the ESC TTE PH probability algorithm we have shown that unsurprisingly increasing PH echo probability is associated with worse RHC haemodynamics. However, despite a TTE multi parameter approach to the assessment of PH, current echo guidelines may not be sufficient to correctly identify those with milder haemodynamic forms of PH. Of those with a high and intermediate probability of PH, RHC PH was identified in 96% and 85% of the respective cohorts. However, in those felt to have a low ESC TTE PH probability, RHC PH was identified in 44% of cases. These cases typically had lower RHC mPAP (23mmHg) when compared to those with intermediate (31mmHg) or high TTE PH probability (41mmHg). Crucially, the mPAP within this low probability group is less than the previous definition of PH (mPAP > 25mmHg). Published literature has also indicated that existing TTE guidelines were insufficient to detect PH [12], therefore, additional echocardiographic techniques are needed to identify patients earlier in the PH cascade.

RV free wall strain is a sensitive marker of RV function [14, 19], and is relatively easy to obtain on modern hardware. We have shown that RVFWS was measurable in 71% of the cohort and its addition using the previously established cut-off of <-23% improved the sensitivity of the ESC PH algorithm from 83% to 92% with similar specificity (65% and 62% respectively). Applying the new cut-offs of -20% in males, and − 21% in females further improved the sensitivity of the algorithm to 93%, but gave a reduction in specificity to 60%. Importantly, a reduced RVFWS was seen with greater frequency in those with a low TTE PH probability than any of the other established markers. This is true of both the previous cut-off of <-23% and the newly published cut-offs for males and females. This group also had a lower mPAP at RHC, suggesting that RVFWS may fall before other geometric and functional abnormalities present as a consequence of advancing PH.

The addition of RVFWS to the ESC TTE PH probability algorithm improved the detection of PH in those felt to have a low echocardiographic probability with sensitivity / specificity improving from 44% / 56% to 81% / 63%, respectively using <-23%.

### Echocardiographic derived PASP

Echocardiographic and RHC-derived PA systolic pressure showed good correlation with one another, however TTE PASP estimates were significantly lower than those for RHC. This is in general agreement with existing work, which demonstrates wide limits of agreement and only moderate precision [7, 20]. Increasing TTE PASP is associated with increasing RHC PASP values (*R* = 0.745, p = < 0.001). However, this study has shown that 1 in 5 of the cohort with PASP < 35mmHg had PH at RHC. Whilst TTE PASP has moderate sensitivity and specificity (75%/73% respectively) for the detection of PH, it cannot be used interchangeably with RHC PASP. Combining TTE PASP with 2 echocardiographic PH markers improved specificity to 82% and if there were > 2 markers present this increased further to 84%. Using TTE PASP > 35mmHg or TRV > 2.8 m/s with at least 2 abnormal TTE parameters (including RVFWS) gave similar sensitivity / specificity (74% / 79% vs. 73% / 87% respectively). It is noteworthy that TTE RAP had particularly poor correlation with RHC PAP and is likely to be the key variable which accounts for the lack of accuracy with TTE PASP estimations.

77% of the whole cohort were classified as intermediate by ESC guidelines for TTE PH assessment, the cut off for which is > 2.8 m/s, or values below this with additional abnormal supporting signs. Our data has shown that the vast majority of patients with confirmed PH and elevated TRV > 2.8 m/s, or TTE-derived PASP have at least 2 abnormal supporting signs of PH (94% and 90%, respectively), and the very small number of patients with abnormal non-invasive haemodynamic pressures with no abnormal ESC markers suggest that with mPAP > 20mmHg it is unlikely to have elevated TRV or PASP without further abnormalities.

When comparing TTE PASP with RHC PASP we have seen that whilst the bias is acceptable, the limits of agreement are wide, emphasising that TTE PASP cannot alone be used on a population basis to screen for the presence of PH. In this cohort TRV was measurable in 86% of patients. In the 14% (*n* = 77) where no TR was measurable, 64% (*n* = 49) had RHC PH.

This agrees with the much of the historical literature which finds TTE PASP to have a good broad agreement, but to be imprecise when applied to individuals due to large under or overestimation in individual instances due to the inherent challenges associated with TR acquisition due to RV geometry [6, 21], as well as the inaccuracy between TTE RAP and RHC RAP [22–24].

### Limitations

This was a single-centre, retrospective study, comprised of a select population of individuals who were referred for PH investigations based on strong clinical history. Therefore, the findings of this study should be assessed in a prospective multi-centre study with a screening population where the prevalence of false positives can be robustly assessed. In addition, given the retrospective nature of the dataset, the images obtained are from several different operators and a variety of different scanning protocols. Screening echocardiography in this group was undertaken following referral into a PH centre and subsequent RHC based on a strong clinical suspicion of PH and consensus following review by a multidisciplinary team of experts, and thus these findings are currently only applicable to patients in whom PH is thought to be present. Further analysis into the impact of pre-test probability and expansion with a control group of patients with no clinical suspicion of PH may help to contextualise the impact of our findings.

RV pacing (RVp) is an established cause of a reduction in cardiac function [25] and whilst only two patients within this cohort had an RV pacing lead in situ, these patients were not excluded prior to analysis. The RVp burden in these individuals is unknown, but they were paced during image acquisition which may have affected RVFWS and accounted for one of the two false negatives in the final analysis. Another additional note is the potential relevance that the period of time between 2020 and 2021 may have on time to RHC, wherein the United Kingdom was under lockdown measures as a result of Covid-19, causing significant disruption to internal services, and is a possible contributing factor to the long time between TTE and RHC.

## Conclusion

We have demonstrated that whilst the current guidelines for the detection of PH an acceptable sensitivity for detection of PH in patients referred for first-time investigation of the condition, almost half of those who were categorised as low probability by TTE were found to have PH at RHC. Those patients with low probability of PH at TTE who received a RHC PH diagnosis had only mildly elevated mPAP, and fewer of the abnormal cardiac markers present within the existing TTE guidelines for the assessment of PH. The addition of RVFWS using both the previously-established cut-off, and the newly proposed values offered in the latest guidelines showed improved detection of these patients.

Current echocardiographic PH algorithms may not be sensitive enough to detect patients with mild elevation of RHC mPAP. Thus, the addition of RVFWS may help to reduce the number of false negatives when compared to current guidelines and assist in expediting those with a low echocardiographic probability of PH and mildly-elevated mPAP to formal diagnosis and subsequent treatment.

## Supplementary Information

Below is the link to the electronic supplementary material.

Supplementary Material 1

## Data Availability

The data that supports the findings of this study can be obtained upon reasonable request from the corresponding author DXA.

## **Abbreviations**

PH: Pulmonary hypertension
TTE: Transthoracic Echocardiography
RHC: Right Heart Catheterisation
mPAP: Mean Pulmonary Artery Pressure
TRV: Tricuspid Regurgitant Velocity
PASP: Pulmonary Artery Systolic Pressure
ESC: European Society of Cardiology
BSE: British Society of Echocardiography
RA: Right Atrium
LV: Left Ventricle
RV: Right Ventricle
TAPSE: Tricuspid Annular Plane Systolic Excursion
PAAT: Pulmonary Artery Acceleration Time
PA: Pulmonary Artery
PV: Pulmonary Valve
RA: Right Atrium
ASE: American Society of Echocardiography
EI: Eccentricity Index
RVFWS: Right Ventricular Free Wall Strain
RAP: Right Atrial Pressure
RVp: Right Ventricular Pacing
EACVI: European Association of Cardiovascular Imaging

## Materials availability

The data that supports the findings of this study can be obtained upon reasonable request from the corresponding author DXA.
