# Supplementary material for: The impact of right ventricular free wall strain on current international echocardiography guidelines for the assessment of pulmonary hypertension
Source: Echo Res Pract. 2026 Jun 8;13:17. doi: 10.1186/s44156-026-00114-6 (PMC13244624; doi:10.1186/s44156-026-00114-6)

**The impact of right ventricular free wall strain on current international echocardiography guidelines for the assessment of pulmonary hypertension.**

**Supplementary analysis**


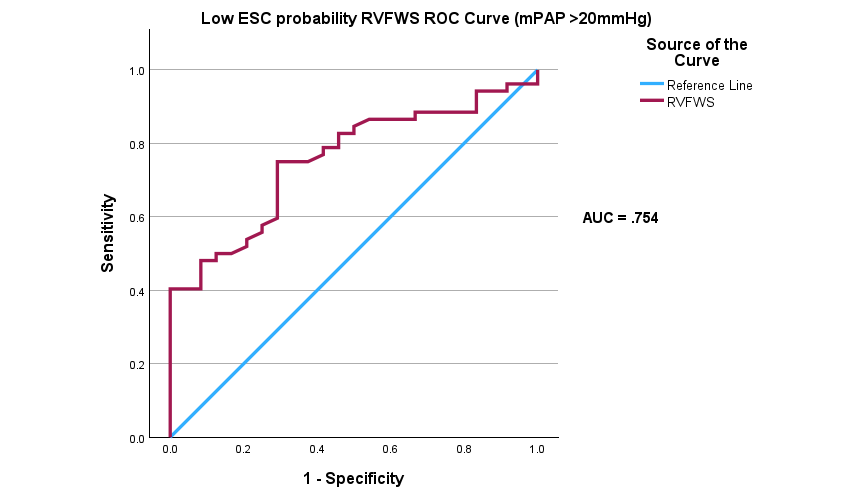

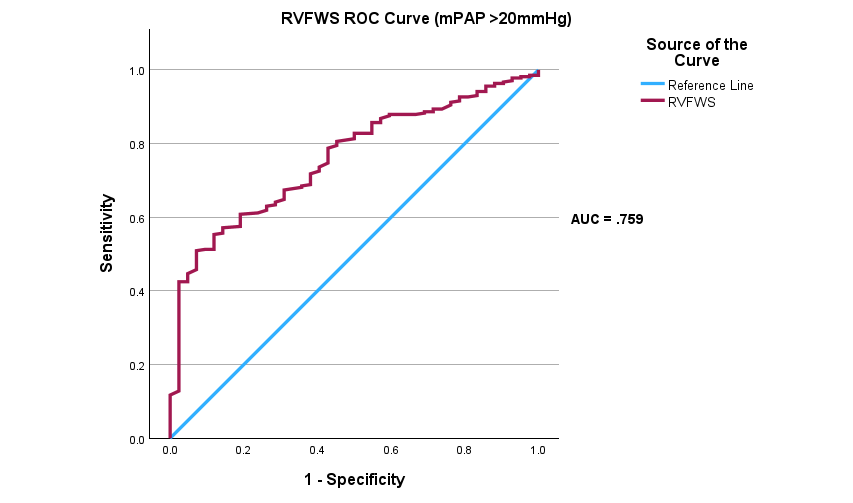

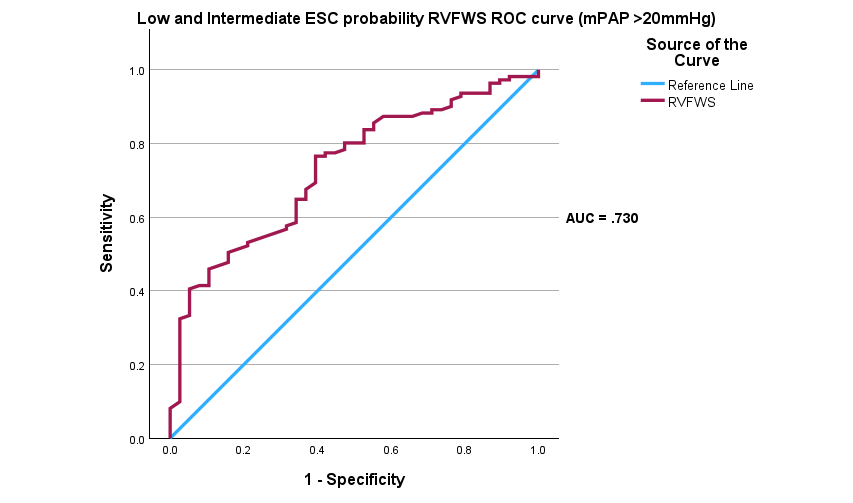


A

B

C

**Supplementary Figure 1:** Receiver Operator Characteristic Curves for the detection of mPAP >20mmHg by RVFWS <-23% in individuals with A: Low TTE-derived probability of PH, B: Low and Intermediate probability of PH, and C: All PH probability tiers as determined by the European Society of Echocardiography guidelines for PH assessment


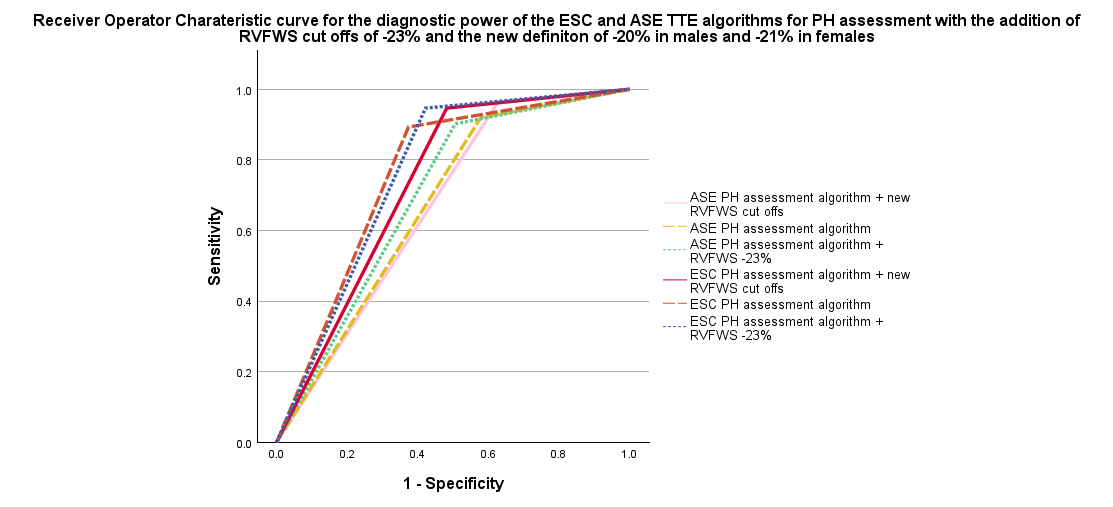


**Supplementary Figure 2:** Receiver Operator Characteristic Curves for the diagnostic power of the ESC and ASE TTE algorithms for PH assessment with the addition of RVFWS cut offs of -23%, and the new definition of -20% in males, and -21% in females.

**Supplementary Table 1:** Receiver Operator Characteristic Curve analysis for the diagnostic power of the ESC and ASE TTE algorithms for PH assessment with the addition of RVFWS cut offs of -23%, and the new definition of -20% in males, and -21% in females.

|  |  | 95% Confidence interval | |
| --- | --- | --- | --- |
| Assessment algorithm | **AUC** | **Lower bound** | **Upper bound** |
| ESC | 0.76 | 0.69 | 0.83 |
| ASE | 0.67 | 0.59 | 0.74 |
| ESC + RVFWS -23% | 0.77 | 0.71 | 0.83 |
| ASE + RVFWS -23% | 0.69 | 0.62 | 0.76 |
| ESC + New RVFWS | 0.73 | 0.66 | 0.80 |
| ASE + New RVFWS | 0.66 | 0.59 | 0.74 |

**Supplementary Figure 3a:** Receiver Operator Characteristic Curves for the European Society of Echocardiography guidelines for PH assessment supporting signs of PH against mPAP >20mmHg in 549 patients referred for first time assessment of PH.


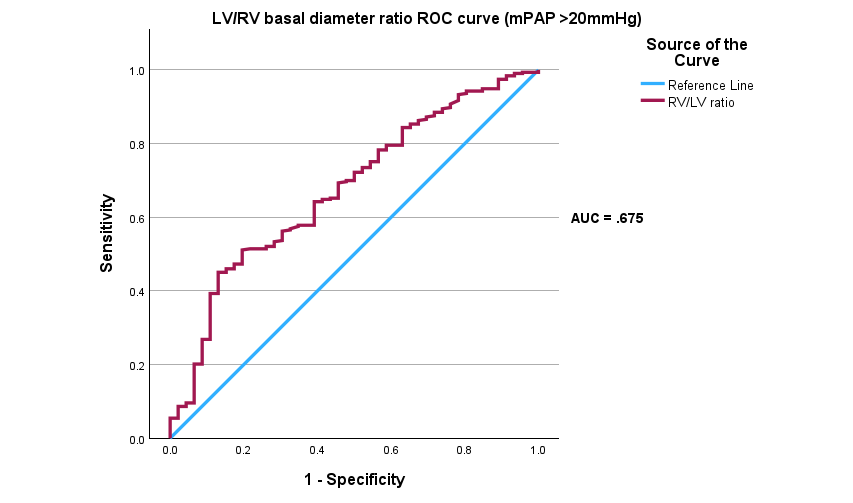

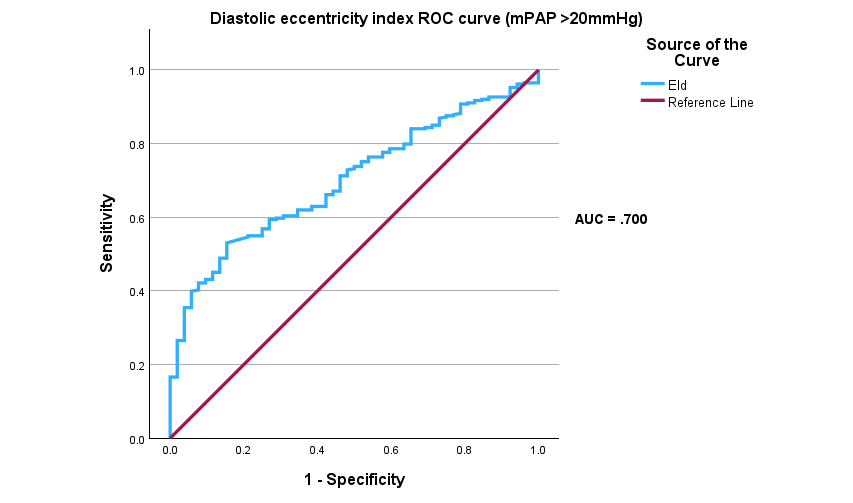

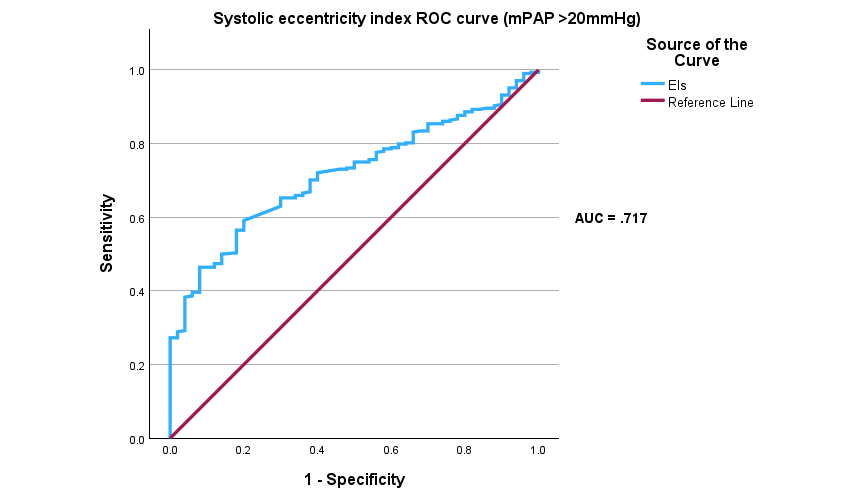


**Supplementary Figure 3b:** Receiver Operator Characteristic Curves for the European Society of Echocardiography guidelines for PH assessment supporting signs of PH against mPAP >20mmHg in 549 patients referred for first time assessment of PH.
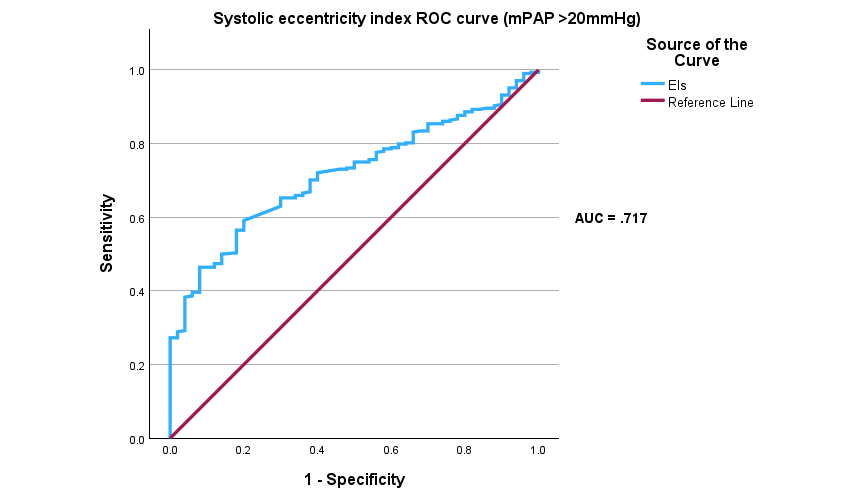

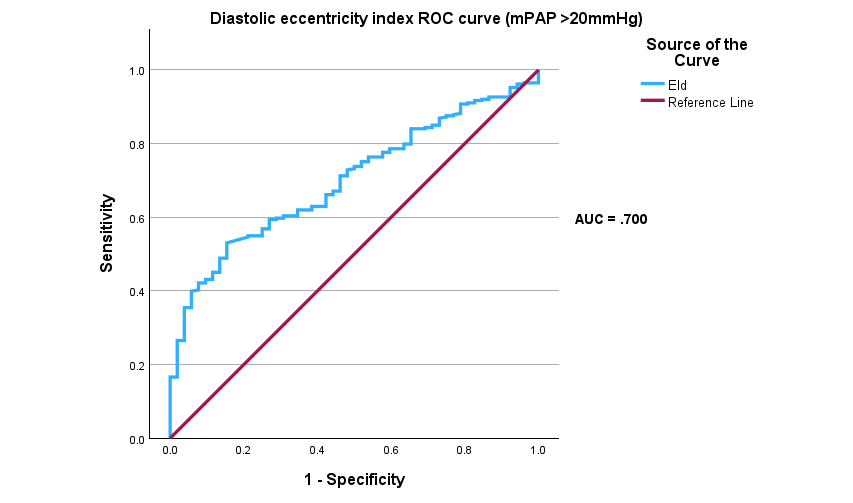


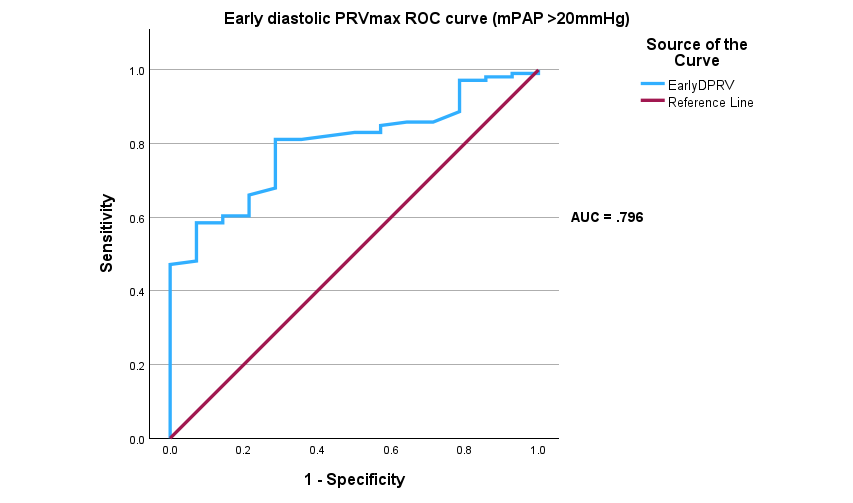

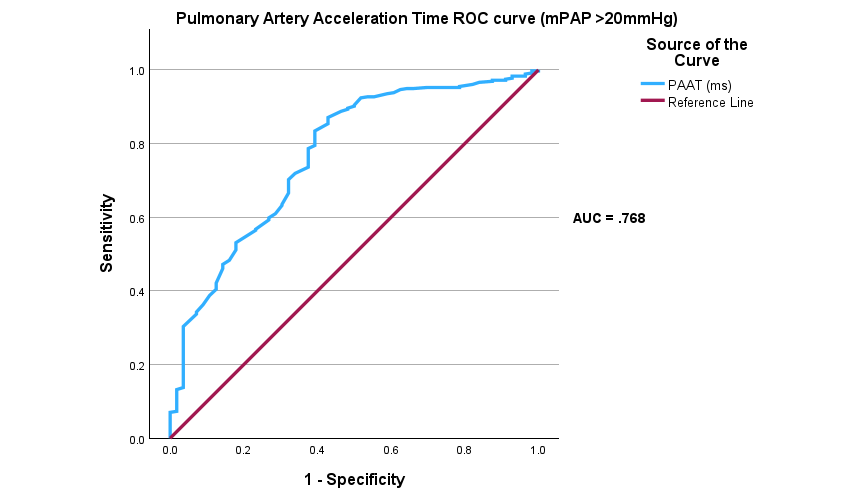

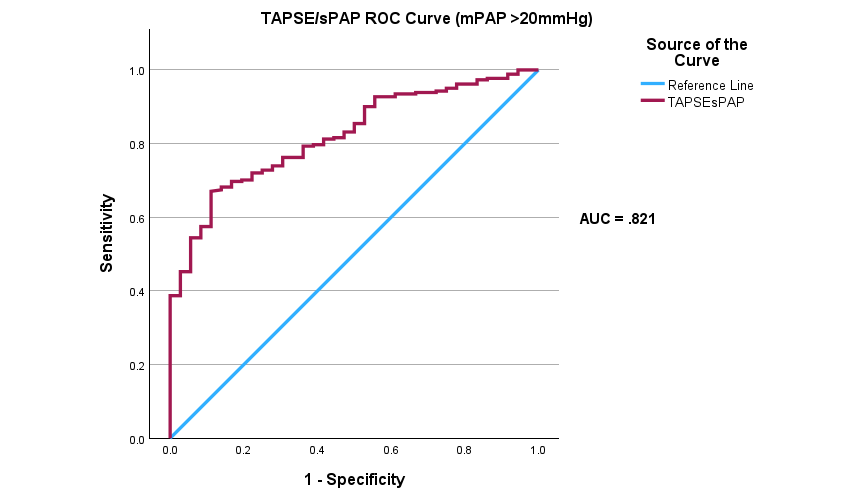


**Supplementary Figure 3c:** Receiver Operator Characteristic Curves for the European Society of Echocardiography guidelines for PH assessment supporting signs of PH against mPAP >20mmHg in 549 patients referred for first time assessment of PH.


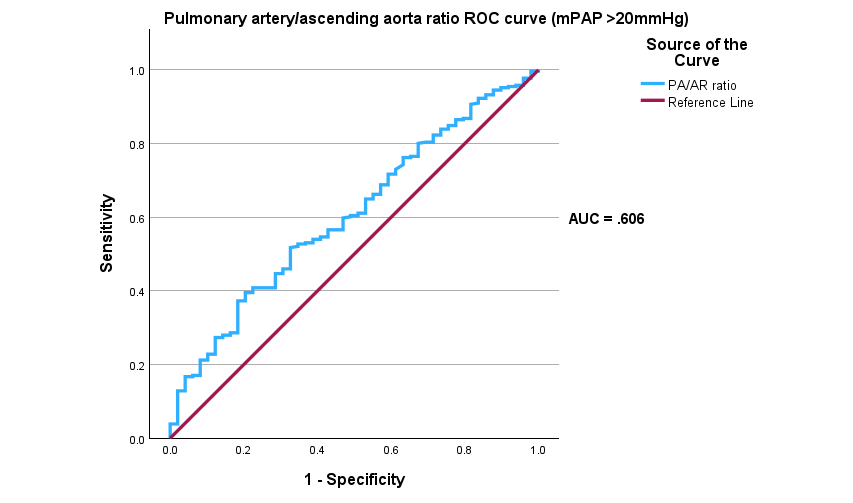

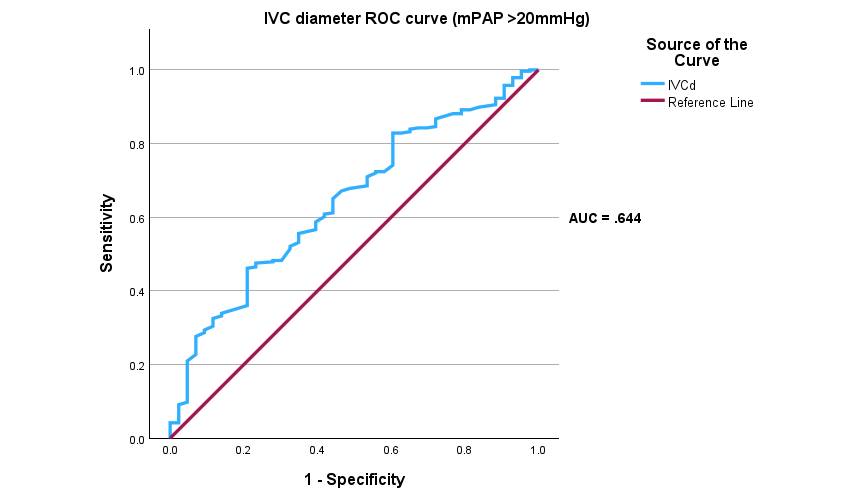

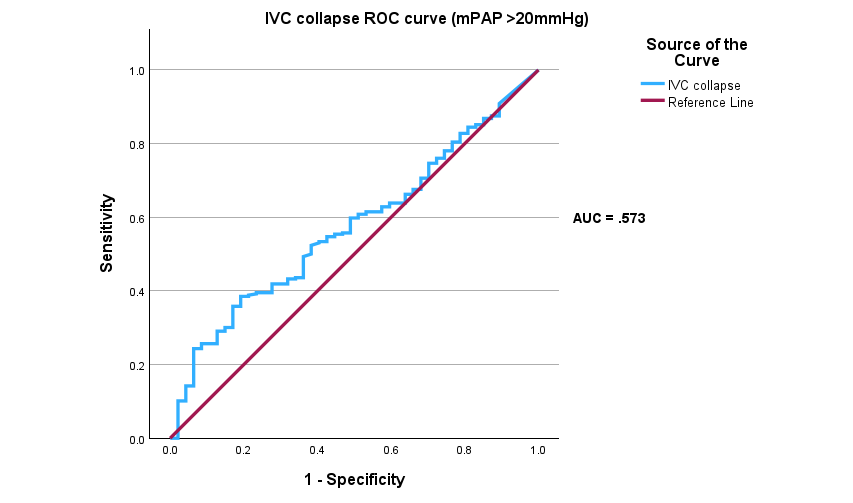

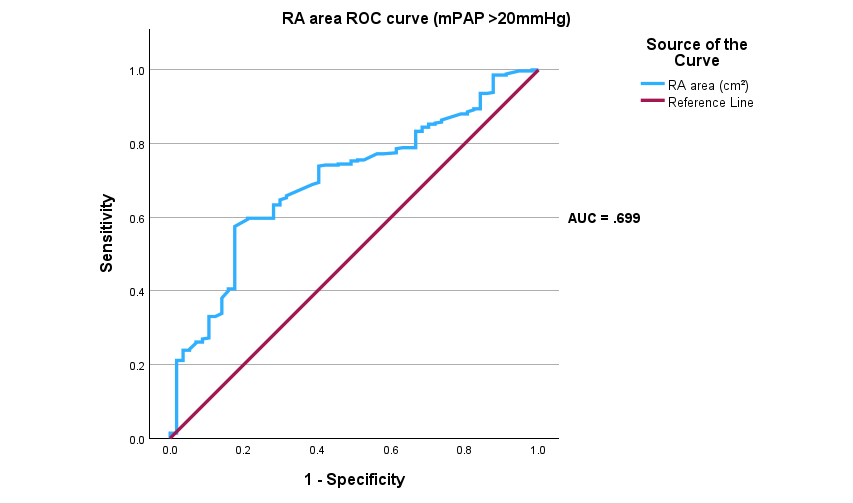

Supplement: Supplementary file 1 — Supplementary Material 1 [file 44156_2026_114_MOESM1_ESM.docx]
